# Supplementary material for: Attitudes towards preventive tuberculosis treatment among hospital staff
Source: PeerJ. 2016 Feb 25;4:e1738. doi: 10.7717/peerj.1738 (PMC4782724; doi:10.7717/peerj.1738)
Supplement: Appendix S1 [file peerj-04-1738-s001.doc]

**Participant Information Sheet**

**Health care worker survey**

Liverpool Hospital

| **Title** | **Attitudes and beliefs among hospital staff towards tuberculosis and preventive treatment** |
| --- | --- |
| **Short Title** | **TB in HCWs** |
| **Project Number** | **12/265** |
| **Investigators** | **Dr Claudia Dobler, Dr Zinta Harrington, Ms Vidya Pathak** |
| **Location** | **Liverpool Hospital** |

**Part 1 What does my participation involve?**

**1 Introduction**

You are invited to take part in this survey, which is called “Attitudes and beliefs among hospital staff towards tuberculosis and preventive treatment”. You have been invited because you work at Liverpool Hospital.

This Participant Information Sheet tells you about the survey. It explains the processes involved with taking part. Knowing what is involved will help you decide if you want to fill in the questionnaire.

Please read this information carefully.

Participation in this research is voluntary. If you don’t wish to take part, you don’t have to.

By filling in the questionnaire you are telling us that you:

• Understand what you have read

• Consent to take part in the survey

• Consent to the use of your personal and health information as described

**2 What is the purpose of this survey?**

As a health care worker at Liverpool Hospital you had to answer questions about your risk of tuberculosis (TB) when you were employed by NSW Health. Maybe you had to go to the chest clinic for some tests. The aim of this screening is to make sure that you don’t have active TB, but also to find out if you have inactive (latent) TB infection. If you have latent TB infection, the chest clinic can offer you preventive treatment to reduce the risk that you get sick from active TB in future.

We are interested to learn more about your experience with our TB screening services and your opinions regarding TB, so that we can improve our service to you in future.

This research has been initiated by the researcher, Dr Claudia Dobler.

**3 What does participation in this research involve?**

We are asking you to fill in a questionnaire, which should take between 5 and 10 minutes to complete.

This questionnaire has been designed to make sure the researchers interpret the results in a fair and appropriate way and avoids study doctors or participants jumping to conclusions.

Return the completed questionnaire in the attached envelope for internal mail. Please do **not** write your name on the survey/response envelope.

**4 Why are you being selected?**

We are asking all nurses, doctors and allied health staff working at Liverpool Hospital to take part in this survey. For staff that work directly with patients screening for TB is important.

**5 Do I have to take part in this research project?**

*.*

Participation in any research project is voluntary. If you do not wish to take part, you do not have to. You may choose not to take the survey, or to skip any questions that you don’t want to answer.

**6 What are the possible benefits of taking part?**

You can tell us what you think about our TB screening services, and what you think we should do differently. We will listen to you and try to improve our screening service to Liverpool Hospital staff.

**7 What happens when the research project ends?**

We will make sure that staff working at Liverpool Hospital will get access to the final results (for example by posting a summary of the survey results on the intranet or by sending out emails to all staff).

**Part 2 How is the research project being conducted?**

**8 What will happen to information about me?**

By filling in the questionnaire you consent to the research team collecting and using the information that you provide in the questionnaire. Any information obtained in connection with this survey is anonymous. Strict confidentiality will be maintained at all times and the information you provide will be protected. Unidentifiable data will be stored on a double password protected computer drive at Liverpool Hospital.

**9 Complaints**

The conduct of this study at **Liverpool Hospital** has been authorised by the South Western Sydney Local Health District, any person with concerns or complaints about the conduct of this study may also contact the Research Governance Officer on (02) 8738 8304, email: [research.support@sswahs.nsw.gov.au](mailto:research.support@sswahs.nsw.gov.au) and quote project number 12/265.

**10 Who is organising and funding the research?**

This research project is being conducted by Dr Claudia Dobler, Dr Zinta Harrington and Ms Vidya Pathak, Department of Respiratory Medicine, Liverpool Hospital, The University of New South Wales.

No member of the research team will receive a personal financial benefit from your involvement in this research project (other than their ordinary wages).

**11 Who has reviewed the research project?**

All research in Australia involving humans is reviewed by an independent group of people called a Human Research Ethics Committee (HREC).

The ethical aspects of this research project have been approved by the HREC of the South Western Sydney Local Health District.

This project will be carried out according to the *National Statement on Ethical Conduct in Human Research (2007)*. This statement has been developed to protect the interests of people who agree to participate in human research studies.

**15 Further information and who to contact**

The person you may need to contact will depend on the nature of your query. If you want any further information concerning this project or if you have any problems which may be related to your involvement in the project, you can contact the researcher Dr Claudia Dobler, ph: 02 87384101, email: [c.dobler@unsw.edu.au](mailto:c.dobler@unsw.edu.au)) or the survey manager (Ms Vidya Pathak, email: v.pathak@student.unsw.edu.au).

**16. Complaints contact person**

This study has been approved by the South Western Sydney Local Health District Human Research Ethics Committee. Any person with concerns or complaints about the conduct of this study should contact the Ethics and Research Governance Office, Locked Bag 7279, LIVERPOOL BC, NSW, 1871 on 02 8738 8304, fax 02 8738 8310, email [research.support@sswahs.nsw.gov.au](mailto:research.support@sswahs.nsw.gov.au), website: <http://www.sswahs.nsw.gov.au/swslhd/ethics/default.html> and quote 12/265.

**Thank you for taking the time to consider taking part in this survey.**

**Questionnaire about attitudes and beliefs among hospital staff towards tuberculosis (TB) and preventive treatment**

| **Health care worker characteristics** | | | | | | | | | | |
| --- | --- | --- | --- | --- | --- | --- | --- | --- | --- | --- |
| 1. Age | □ ≤30 □ 31-40 □ 41-50 □ >50 | □ 31-40 | | | □ 41-50 | | | □ >50 | | |
| 1. Sex | □ male | □ female | | |  | | |  | | |
| 1. Profession | □ doctor  □ nurse | | | | □ allied health  □ other | | | | | |
| 1. Do you have direct patient contact at work? | □ yes | | | | □ no | | | | | |
| 1. Please provide your department/ward | □ respiratory medicine  □ other medical  □ surgery  □ O&G  □ paediatrics | | | | □ ED  □ ICU  □ laboratory/pathology  □ other | | | | | |
| 1. What country were you born in? | ………………………………………….. | | | | | | | | | |
| 1. If you were born in Australia, have you stayed overseas for ≥ 3 months in the past? | | □ yes | | | □ no | | |  | | |
| **Past experience with TB screening** | | | | | | | | | | |
| 1. Have you had a BCG vaccination? | | | □ yes | | | □ no | | | □ don’t know | |
| 1. Have you undergone TB screening for health care workers in the past? | | | □ yes | | | □ no | | | □ don’t know | |
| 1. If yes (q9), was the screening done at Liverpool Hospital Chest Clinic? | | | □ yes | | | □ no | | | □ don’t know | |
| 1. When was the screening performed? | | | □ after Feb 2011 | | | □ 2007 to Feb 2011 | | | □ prior to 2007 | |
| 1. Have you had a chest x-ray as part of TB screening? | | | □ yes | | | □ no | | | □ don’t know | |
| 1. If yes (q12), do you know the result? | | | □ yes | | | □ no | | |  | |
| 1. Have you had a Tuberculin Skin Test (TST) as part of TB screening? | | | □ yes | | | □ no | | | □ don’t know | |
| 1. If yes (q14), did you have a positive reaction? | | | □ yes | | | □ no | | | □ don’t know | |
| 1. Have you had a TB blood test (QuantiferonGOLD) as part of TB screening? | | | □ yes | | | □ no | | | □ don’t know | |
| 1. If yes (q16), did you have a positive result (indicating TB infection)? | | | □ yes | | | □ no | | | □ don’t know | |
| 1. If you had a TST or TB blood test (Quantiferon GOLD) in the past, do you feel you were provided with adequate information about the result? | | | □ yes | | | □ no | | | □ not applicable | |
| 1. If you had a positive TST or a positive blood test for latent TB (QuantiferonGOLD) in the past, were you offered preventive treatment for latent (dormant) TB infection? | | | □ yes | | | □ no | | | □ not applicable | |
| 1. If yes (q19), did you take preventive treatment? | | | □ yes | | | □ no | | |  | |
| 1. If no (q20), why not? | | |  | | | | | | | |
| □ possible side effects  □ negative consequence for job  □ I have been vaccinated against TB  □ other | | | □ treatment is unnecessary  □ treatment is ineffective  □ I can keep an eye on whether I develop symptoms of TB | | | | | | | |
| 1. Do you know the difference between active TB and latent (dormant) TB infection? | | | □ yes | | | □ no | | |  | |
| 1. Did you receive adequate information about your TB status? | | | □ yes | | | □ no | | |  | |
| 1. Have you had TB in the past? | | | □ yes | | | □ no | | | □ don’t know | |
| **Personal opinions** | | | | | | | | | | |
| 1. Do you think that hospital staff who has evidence of latent (dormant) TB infection, should receive preventive treatment in general (usually daily tablets for 6-9 months)? | | | | □ yes | | | □ no | | | □ no opinion |
| 1. If you had latent (dormant) TB infection, would you like to be offered preventive treatment (usually daily tablets for 6-9 months)? | | | | □ yes | | | □ no | | | □ no opinion |
| 1. Do you feel you would need more information to make this decision (q26)? | | | | □ yes | | | □ no | | | □ no opinion |
| 1. Do you think staff born in Australia without prolonged overseas stay should be tested for TB even though they are at very low risk of developing TB? | | | | □ yes | | | □ no | | | □ no opinion |

Thank you for participating in this survey!
